# Leveraging learning experience design: digital media approaches to influence motivational traits that support student learning behaviors in undergraduate online courses

**DOI:** 10.1007/s12528-022-09342-1

**Published:** 2022-10-11

**Authors:** Joseph T. Wong, Bradley S. Hughes

**Affiliations:** 1 University of California, 3200 Education Bldg, Irvine, CA 92697 USA; 2 University of California, Steinhaus Hall, Irvine, CA 92697 USA

**Keywords:** Learning experience design, User experience design, Instructional design, Motivation, Learning behaviors, Digital Media

## Abstract

Higher education may benefit from investigating alternative evidence-based methods of online learning to understand students’ learning behaviors while considering students’ social cognitive motivational traits. Researchers conducted an in situ design-based research (DBR) study to investigate learner experience design (LXD) methods, deploying approaches of asynchronous video, course dashboards, and enhanced user experience. This mixed-methods study (*N* = 181) assessed associations of students’ social cognitive motivational traits (self-efficacy, task-value, self-regulation) influencing their learning behaviors (engagement, elaboration, critical thinking) resulting from LXD. Social cognitive motivational traits were positively predictive of learning behaviors. As motivational factors increased, students’ course engagement, usage of elaboration, and critical thinking skills increased. Self-efficacy, task-value, and self-regulation explained 31% of the variance of engagement, 47% of the explained variance of critical thinking skills, and 57% of the explained variance in the usage of elaboration. As a predictor, task-value beliefs increased the proportion of explained variance in each model significantly, above self-efficacy and self-regulation. Qualitative content analysis corroborated these findings, explaining how LXD efforts contributed to motivations, learning behaviors, and learning experience. Results suggest that mechanisms underpinning LXD and students’ learning behaviors are likely the result of dynamically catalyzing social cognitive motivational factors. The discussion concludes with the LXD affordances that explain the positive influences in students’ social cognitive motivational traits and learning behaviors, while also considering constraints for future iterations.

## Introduction

Higher education may have significant potential to identify practical ways to improve undergraduate online learning experiences through the novel combination of Learner Experience Design (LXD), educational technologies, and testing through design-based research (DBR). This study was afforded by the rare, rapid and massive conversion to distance learning platforms implemented during the COVID-19 pandemic. Through the sudden immersion into an array of online learning implementation, students and learning experience designers are afforded a unique opportunity to rapidly examine which technology based pedagogical approaches are most effective, based on interventions and observations made within the natural large-scale higher educational settings. The accelerated conversion to online learning provided a unique teaching and learning context, opening a useful window into investigating the applications of online LXD for the study of computers in higher education.

Although it may be commonplace to utilize the popular synchronous “Zoom internet-mediated teleconferencing method” for online learning (Chick et al., 2020; Verma et al., 2020), to the contrary, a LXD investigator might hypothesize higher efficacies from designing an asynchronous self-paced online course that integrates the combination of pedagogical designs and user interface design. However, considering the significant technological, pedagogical, and training demands involved (Rapanta et al., 2020), it may be no surprise that online learning has traditionally been slow to take hold in universities. Taking a design-based research (DBR) approach, this study investigated an online instructional course that enabled researchers to evaluate student interactions in situ*,* in both real-world settings and extreme situations (Collins et al., 2004; Siek et al., 2014). The COVID-19 pandemic necessitated a systematic change in university course delivery (Agarwal & Kaushik, 2020; Ferrel & Ryan, 2020). Consequently, when presented with the rapid transition to remote learning, we quickly conducted a DBR study deploying an instructor-designer developed asynchronous self-paced online course grounded in LXD to collect empirical data in the wild. This notion of “in the wild,” is in reference to the naturalistic usage of introducing a novel design in the field and performing extended evaluations within the intended population and context of use (Siek et al., 2014).

The quality of online courses may vary markedly, due to insufficient training from instructors (Hodges et al., 2020) and the unwillingness of institutions to adopt digital learning tools (Rapanta et al., 2020). While online learning can be an effective way to foster teaching and learning (Mayer & Moreno, 2010; Muljana & Luo, 2020; Taipjutorus, 2014; Xu & Xu, 2020; You, 2016), many institutions are placing more attention on the expeditious transfer of the same in-person educational content into synchronous teleconferencing lectures in the online learning environment, rather than developing online courses grounded in evidence-based instructional designs and teaching pedagogies. When learning online, students are required to adapt to different learning contexts and modalities, potentially affecting their motivations and learning behaviors within the online environment. In a recent study, Adnan and Anwar (2020) found that 71.4% of undergraduate students reported that learning in conventional face-to-face classrooms was more motivating than distance learning. Additionally, Rapanta et al. (2020) argue that instructors not only face the technical struggles of delivering online instruction, but also lack the instructional and pedagogical training necessary to “design and administer meaningful online learning experiences.” Furthermore, learners spending more time worrying about accessing, locating, and finding course content within the user interface are likely to experience greater frustration and confusion within an online learning environment (Hu, 2008; Shneiderman & Hochheiser, 2001). This combination of low student motivation, poor instructional grounding in learning design, and overlooking the learner’s user experience have led to undergraduate learners citing issues of diminished engagement, poor time management, and low levels of confidence with their own abilities to learn online, primarily due to their unfamiliarity and lack of prior experience with online courses (Agarwal & Kaushik, 2020; Sun & Rueda, 2012; Tullis & Benjamin, 2011; Zayapragassarazan, 2020). Thus, to support students' learning behaviors, attention may be shifted advantageously to include evidence-based principles of learning experience design, with a focus on monitoring factors including students’ self-efficacy, task-value, self-regulation, engagement, elaboration, and critical thinking. This presents a challenge and opportunity for the development of online courses that experiment with approaches that go beyond merely duplicating in-person lectures into online spaces through Zoom teleconferencing, and explore DBR approaches to online teaching that leverage the expanding digital learning media tools available to online course designers.

## Theoretical background

### Online learning

Over the last two decades, there has been growing interest in online courses supporting student teaching and learning, particularly for their flexibility, convenience, and the ability to reach more isolated populations (El Ahrache et al., 2013; Marrongelle et al., 2013; Cetina et al, 2018). Online learning facilitates learning partially or entirely over the internet (Means et al., 2009; Richardson & Newby, 2010), through synchronous and asynchronous modalities. Looking at MOOCs (Massive Open Online Courses), course platforms such as Udemy and Coursera offer online degrees and certificates over an asynchronous delivery platform, facilitating online self-paced learning (Cetina et al., 2018). Conversely, synchronous learning requires students to be present during an allotted time, emphasizing the social presence between teachers and students through teleconferencing (Cobb, 2009; Jaggars & Xu, 2016; Means et al., 2009; Xu & Xu, 2020). Compared to synchronous courses, asynchronous self-paced courses have been shown to foster increased learner independence, individualized instruction, personal responsibility, review and practice, and increased test preparation (Alqurashi, 2016; Holmberg, 2003; Morris et al., 1978; Richardson et al., 2016). Furthermore, Tullis and Benjamin (2011) argue that when learners actively engage in their own productive metacognitive judgments at their own pace, students are more likely to succeed in online learning environments by monitoring their study-time allocation, self-agency, and motivational traits. However, these successful skill-building learning outcomes in online courses are attributed to careful considerations of learning experience design. Without quality learning experience design, students are more likely to feel disengaged, lose motivation, and oftentimes fail to complete the online course (Czerkawski & Lyman, 2016; You, 2016). Thus, in order to better support students to develop these skills, actively engage in their coursework, and achieve high completion rates, online courses might be transformed to be grounded in evidence-based teaching pedagogy and learning experience design principles.

### Learning experience design

Learning experience design (LXD) refers to the creation of learning situations that extend beyond the formal classroom learning environment and which often utilize online and educational technologies (Ahn, 2019). Coined as a term in 2015, LXD is the process of developing effective learning experiences that enable learners to reach a specified learning outcome in a human-centered goal-oriented method (Floor, 2018). LXD is a departure from the traditional term “instructional design,” which is primarily focused on curriculum development and programming instruction to support knowledge acquisition (Correia, 2021). More specifically, instructional design refers to the systematic approach of delivering effective instruction for learners with the goal of reaching high levels of achievement and consuming information (Branch & Merill, 2012; Joo et al., 2015). Conversely, Weigel (2015) further defines LXD as an interdisciplinary synthesis of instructional design, teaching pedagogy, cognitive science, learning sciences, social science, and user experience design (UXD). In practice, Floor (2018) defines the five fundamentals of LXD as human-centered, goal-oriented, based upon theory of learning, including learning through practice, and being heavily interdisciplinary. In each of these five facets, there is a major emphasis on empathy, focusing on the intended and unintended design outcomes for the learners (Matthews et al., 2017). As such, LXD broadens our definitions of what is to be considered a learning experience, affording instructors, designers, and researchers the opportunity to empathize with learners and develop experiences that expand our design toolbox to support students’ motivation as well as learning behaviors in diverse learning settings (Ahn et al., 2019; Weigel, 2015).

### Situated cognition theory

In the context of this study, operationalizing our LXD first involved grounding the online course in a pedagogical learning design framework. Designing online courses with the Situated Cognition Theory (SCT) has been shown to facilitate students’ interests (Ghefaili, 2003) and may inform the design of effective e-learning experiences while students acclimate to distance learning (Cakmakci et al., 2020). Centered around the notion that “learning” is inseparable from “doing,” this framework was adopted so that learners could grasp the concepts and skills that are taught in the context in which they will be utilized (Brown et al., 1989; Collins et al., 1991). In practice, SCT emphasizes immersive learning environments, where new information is taught to learners in a way that simulates real-life settings. Applying this methodology involved using high-quality 4 K multi-camera green screen video production integrated with lecture hall-style presentation graphics integrating natural realism and animated notations. In addition to teaching with video, the new online course version included an array of digitally interactive tools by embedding opportunities for modeling, coaching, scaffolding, articulation, reflection, and exploration (Collins et al., 1991; Pappas, 2015). Through this framework, students watched bite-sized video scaffolded segments of the instructor teaching concepts of evolutionary psychology while making use of the green screen to foster an immersive learning environment. Strategically placed between the video scaffolds were engagement questions, reflective prompts, and discussions that were designed for students to immediately practice what they had just learned. As such, the SCT e-learning design framework was implemented to foster a scaffolded learning experience for students to actively develop confidence in their learning experience, provide a systematic routine for online learning, and to metacognitively engage within the online learning environment.

### Task-value and self-efficacy

Introducing a video scaffold design may further support learners’ perception and confidence while learning remotely. Social cognitive theorists of behavior and motivation link task-value and self-efficacy as significant predictors for students’ online learning success, where an individual’s perceived judgments influence the learners’ action-outcome expectancy (Artino & McCoach, 2008; Albert Bandura, 1977; Pintrich, 1999; Skaalvik & Skaalvik, 2010). Eccles and Wigfield (2002) define task value in four components: attainment value as the importance of doing well; intrinsic value as a person’s subjective interests; extrinsic value as perceived usefulness; and cost as associated with negative aspects for participating. In practice, task-value references students’ perceived interests, importance, and usefulness while participating in a learning task (Pintrich, 1999). Past research has found that when students participate in learning activities that actively develop such value components, students are more likely to develop and solidify their involvement in the course (Chen et al., 2001; Joo et al., 2015). Such increased involvement activated by the learning environment may serve as a powerful motivator when learner participation and interaction throughout the entire learning process are sustained. Leveraging the SCT framework for e-learning course design, our scaffolded approach segments videos and affords opportunities for teacher-guided instruction and learner-centered participation. By fostering a deeply structured situated learning environment for students to actively develop interests and values contextually within the online learning environment, we assess students’ task-value as a result of our LXD efforts.

Bandura’s (1977) framework of self-efficacy states that people will perform an action if they are motivated and confident that their behavior will have a favorable outcome (Bleicher, 2004; Schunk, 2006). Similarly, student self-efficacy in an online course refers to the confidence in their own abilities to learn successfully and the action-outcome expectancy students take in an online learning environment (Pintrich, 1999; Bandura, 2000). This self-appraisal of one’s ability to perform a task based on his or her own judgments has been recognized as a critical factor that influences student achievement and behavior (Skaalvik & Skaalvik, 2010; Tschannen-Moran & Hoy, 2001). Corroborating results from multiple studies, researchers have found that students who held higher online learning efficacy beliefs were more motivated, engaged, and exhibited increased learner control (Alqurashi, 2016; Caldwell et al., 2010; Taipjutorus, 2014). Ketelhut (2007) also found that students with high self-efficacy beliefs were more likely to persevere when encountering obstacles and difficult learning situations. Moreover, recent studies have shown that self-efficacy and task-value are linked with students’ online learning strategies such as increased engagement, critical thinking (applying previous knowledge to new situations) and use of elaboration (summative aligning of new information) (Ali et al., 2014; Artino & McCoach, 2008). Wang et al. (2013a) found that when students held higher levels of motivation while distance learning, their course satisfaction and learning strategies increased, resulting in better performance in course outcomes in online settings. Aligning with this evidence and to target these skill-building learning strategy outcomes, our LXD video scaffolded approach was employed to systematically guide students to develop their personal confidence in their abilities in order to successfully execute their learning outcomes (Pellas, 2014; Zhang & Lu, 2002).

### Self-regulation

Due to the self-paced nature of the course design, developing students’ self-regulation skills throughout the online learning environment was an important factor in improving success for learners. Self-regulation refers to the human’s ability to monitor or manipulate their thoughts and actions to reach a specific objective (Pellas, 2014; Vrugt & Oort, 2008; Zimmerman & Schunk, 2001). More specifically, self-regulation in an online course is defined as the extent to which students elicit self-regulated metacognitive skills and strategies during a learning activity in order to be successful in an online course (Wang et al., 2013a, 2013b; Wolters et al., 2006). Contrary to face to face in-person instruction, asynchronous self-paced online courses inherently lack the physical presence of an instructor directly facilitating and guiding instruction. While the instructor may be present through the videos posted, students do not physically present themselves or interact in a face-to-face context through an asynchronous course. However, the benefits of implementing an asynchronous online course provide learners autonomy, with the choice of deciding when to learn, where they want to access the materials, and for how long (McMahon & Oliver, 2001; Wang & Lin, 2007). Although this might be a significant shift in responsibility to the learner when compared to traditional instruction, instructors and designers can support students’ self-regulation skill training by explicitly instructing students to monitor their own thinking process, setting proximal and distal goals, allocating enough time to accomplish assignments, digital interactivity, usability descriptions, and proper scaffolding (Al-Harthy et al., 2010; Kanuka, 2006; Shneiderman & Hochheiser, 2001). In doing so, students actively use many cognitive and metacognitive strategies to manipulate, control, and regulate their own learning behaviors to accomplish the required tasks (Wang et al., 2013a, 2013b). By incorporating these LXD choices, the course may better support students’ online self-regulation skills in order to further facilitate students’ learning behavior strategies such as engagement, elaboration, and critical thinking while distance learning.

### Student learning behaviors

In this study, three types of student learning behaviors, which include engagement, use of elaboration, and critical thinking skills, are discussed in this LXD-based distance learning context. In education, student engagement is defined as the amount of student effort or active participation needed to complete a learning task (Hu & Kuh, 2002; Richardson & Newby, 2010). In an online course, engagement can be further described as the attention, curiosity, interactivity, and interest that students exhibit during an instructional unit, which further extends to the level of motivational traits that students may utilize during the learning process (Pellas, 2014). Engagement has been found to have a significant and positive relationship with student outcomes such as students’ progress in learning, course satisfaction, and course grades (Bolliger & Halupa, 2018). When online courses are not grounded in learning theory, or they are difficult to navigate, uninteresting, or unengaging, studies have shown that this will likely lead to negative course engagement behaviors such as increased mind-wandering, or the directing of attention away from a primary task (Desideri et al., 2019). Elaboration strategies are students’ ability to store information into their long-term memory through the summative aligning of conceptual content and activities (Pintrich et al., 1993). These activities are considered to be “meaningful and sensemaking” which include tasks such as summarizing, generative note taking, analogical reasoning, and mental representations of new conceptual information learned (Weinstein, 1986). Social cognitive researchers have linked student’s self-efficacy and task-value to positive predictions in students’ usage of elaboration in distance learning environments (Ali et al., 2014; Artino & McCoach, 2008). Moreover, elaboration has also been consistently predictive of greater student achievement, especially when students move away from shallow processing strategies such as merely underlining or mechanically memorizing information (Greene et al., 2004; Pintrich et al., 1993). On the other hand, critical thinking is the ability for students to apply new and prior knowledge of conceptual content and derive decisions based on the evaluation of that content (Pintrich et al., 1993). Student-generated activities might include searching for multiple sources of representations, critically questioning information, and making assessments based on this information to draw conclusions (Uzuntiryaki-Kondakci & Capa-Aydin, 2013). More specifically, Brookfield (1987) defines critical thinking in the context of research, as the recognition of the learners’ assumptions that underpin their thoughts and actions. As students perform critical thinking behaviors in a learning environment, research has found that critical thinking requires learners to metacognitively monitor their own thoughts, reactions, perceptions, assumptions, and confidence in the material (Bruning, 2005; Halpern, 1998; Jain & Dowson, 2009; Wang et al., 2013a, 2013b). This indicates that students’ critical thinking skills may be influenced by students’ self-efficacy, task-value, and self-regulation. Furthermore, fostering students’ critical thinking skill building may also support their transferable skills (Fries et al., 2020), a key competency for STEM undergraduate students at R1 institutions linking course materials to real-world applications.

Research in online learning attributes increased student learning behaviors due to quality instructional design (Marrongelle et al., 2013; Pappas, 2015), learner experience within the course user interface (Floor, 2018; Hu, 2008), and student social cognitive motivational factors (e.g. task-value, self-efficacy, self-regulation) that can emerge as a result of the learning environment (Artino & McCoach, 2008; Belcheir & Cucek, 2001; Sun & Rueda, 2012). By implementing LXD, we take a human-centered empathetic lens and attune our course designs to better account for students' diverse learning conditions and changing learning behaviors (Ahn et al., 2019; Xie, 2020). For example, we designed the online modules to be flexible and learner-paced, enabling students to start on their own time and work through the course at their own speed (Richardson & Newby, 2010). Additionally, opportunities for engagement, elaboration, and critical thinking were maximized through the inclusion of virtual coaching, scaffolded videos, and metacognitive journal reflections embedded within each weekly module that facilitated students in more sustained participation and interactivity. Fink (2007) writes, “when course design models are used to restructure the learning experience, as a response, students become more actively engaged in the learning process because the intended learning holds greater meaning.” Thus, LX course design efforts were adopted to better elicit students’ motivations in order to further support students’ learning behaviors while distance learning.

### Current study

**Fig. 1:**
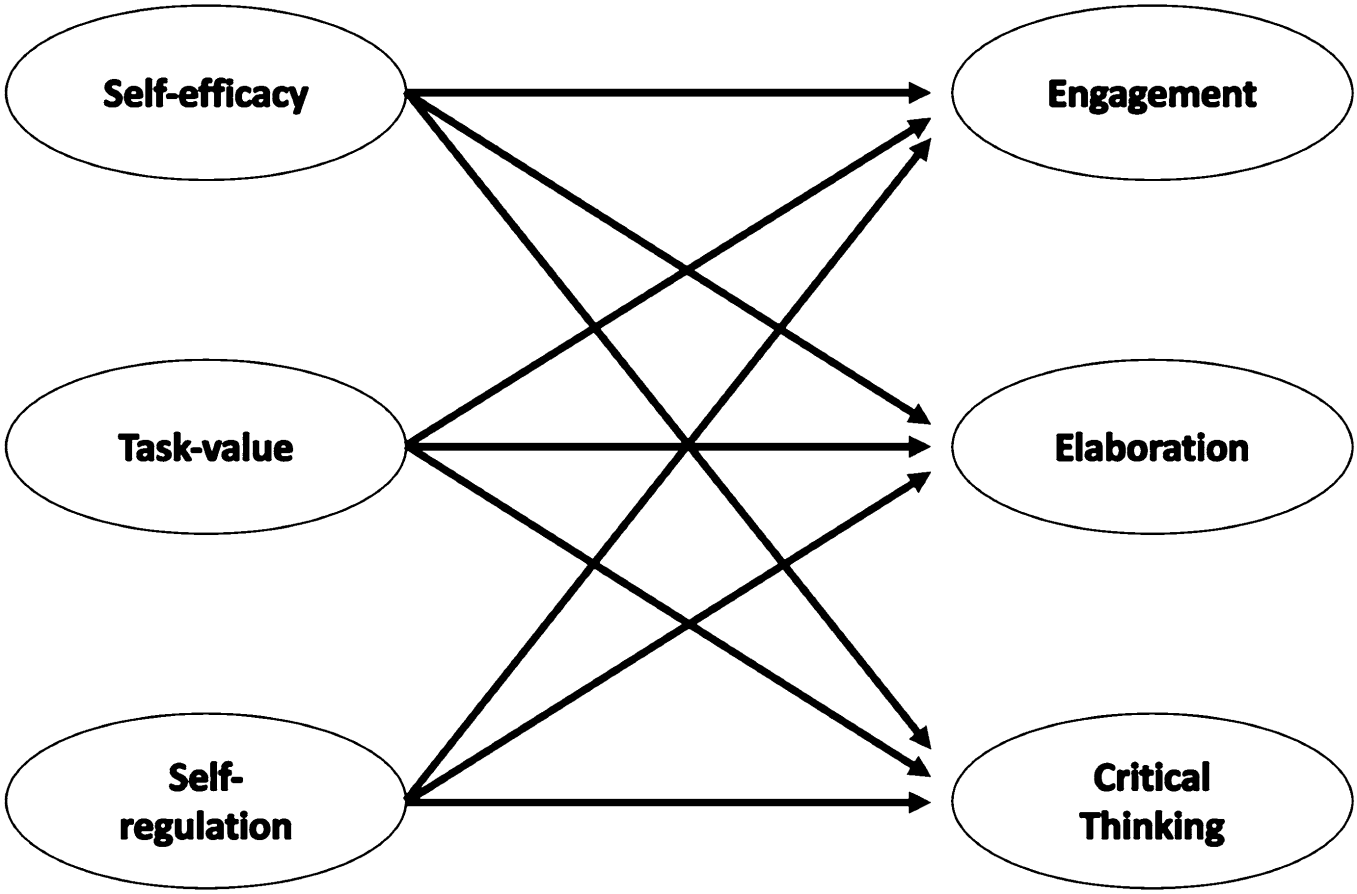
Model of research questions

Little has previously been reported about how online learning with a learning experience design (LXD) approach may affect students' social cognitive motivational factors and students’ learning behaviors. As such, we designed an online course for the purpose of increasing students' social cognitive motivations and learning behaviors by grounding the online learning environment in LXD. The LXD was operationalized by aligning the online course with the SCT pedagogical framework, producing segmented animated video scaffolds, and implementing user experience design heuristics to create affordances that directly support students empathetically. To our knowledge, this integration of learning design and user experience is a relatively new field of education in its infancy and this study sought to better understand how undergraduate students’ personal social cognitive motivational factors (self-efficacy, task-value, self-regulation) support or hindered their online learning behaviors (engagement, elaboration, critical thinking) as a direct result of the LXD efforts (See Fig. 1).

Thus, this study is guided by the following research questions:

1. To what extent do students’ online self-efficacy, task-value, and self-regulation influence students’ perceived online engagement while learning in an online environment?
2. To what extent do student’s self-efficacy, task-value, and self-regulation influence students’ elaboration learning behaviors while learning in an online environment?
3. To what extent do student’s self-efficacy, task-value, and self-regulation influence students’ critical thinking learning behaviors while learning in an online environment?
4. To what extent did the LXD approach (learning and user experience design considerations) support students’ online learning experience?

## Methodology

### Participants

Participants in this study included undergraduate students from a large R1 university in California within the School of Biological sciences. There were a total of 207 undergraduate students enrolled in two separate sections of the lower division general education elective Evolutionary Psychology online science course. Out of the 207 students enrolled, (*N* = 181) students responded to both the pre and post-assessments, representing a survey response rate of 87.4%. These undergraduate students were of varying student-level statuses, with 42.6% first year, 21.3% second year, 12.0% third year, 20.2% fourth year, and 3.8% fifth year students enrolled (See Table 1). The demographics of students in this study were 2.76% African American, 48.6% Asian/ Pacific Islander, 29.8% Hispanic, 12.3% white, and 6.14% other ethnic/ racial groups, comprised of (*N* = 121) females, and (*N* = 60) males (See Table 1). Additional student demographic data is provided in Table 1.

**Table 1:** Sociodemographic characteristics of participants

Student characteristics	Students enrolled	
	*n*	%
Gender		
Female	121	66.1
Male	60	33.9
Ethnicity		
African American	5	2.76
Asian	88	48.6
Hispanic	54	29.8
White	23	12.7
Other	11	6.14
Student Year		
First	78	42.6
Second	39	21.3
Third	22	12.0
Fourth	37	20.2
Fifth	5	3.80
Underrepresented minority		
Yes	79	43.6
No	102	56.4
First generation		
Yes	104	57.4
No	77	42.6
Low income		
Yes	84	46.4
No	97	53.6

*N* = 181

### Design-based research context

**Fig. 2:**
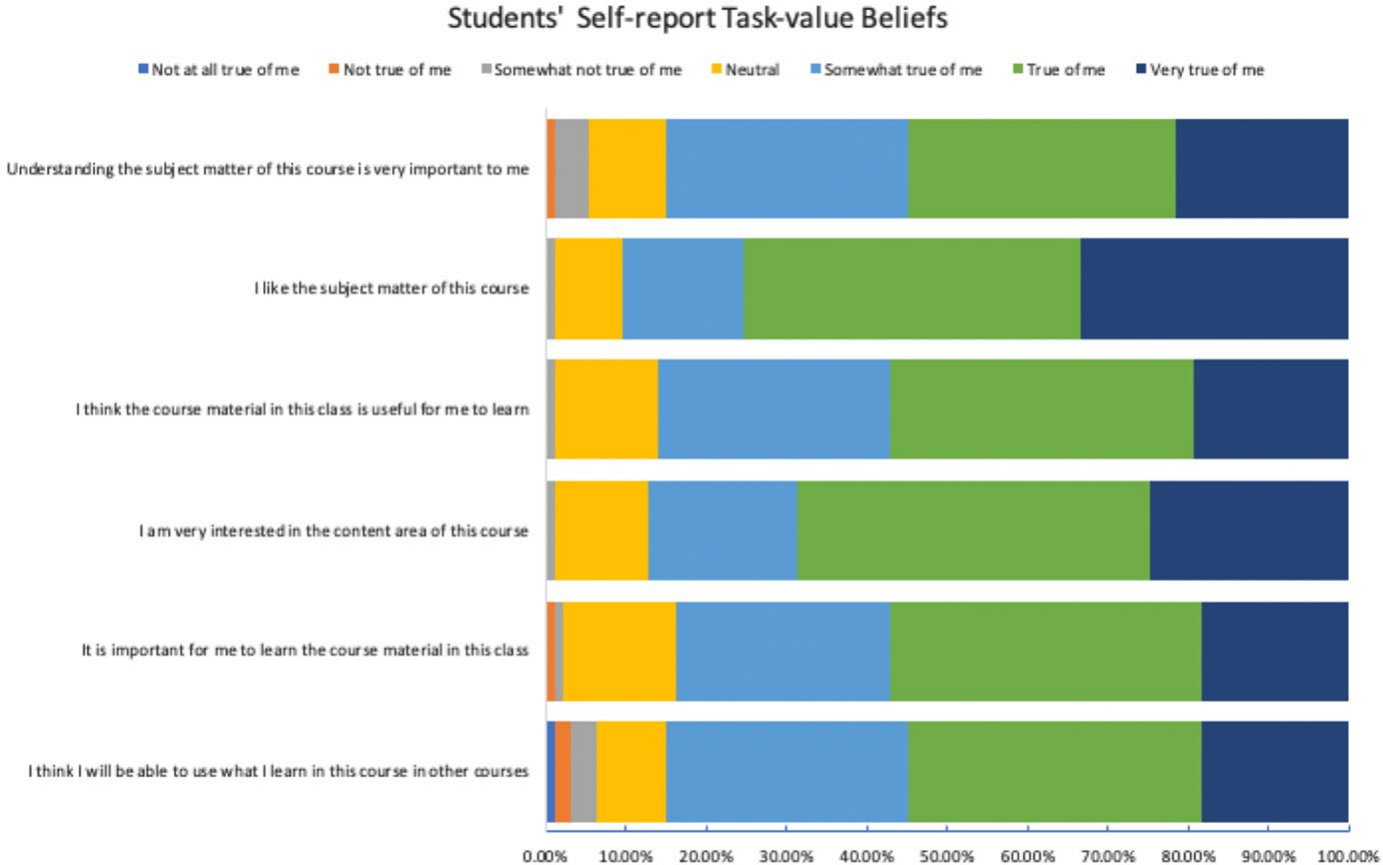
Students’ self-report task-value beliefs at the end of the academic quarter. Response values were normalized to percentages and stacked horizontally for visual representations

This study employed an in situ design-based research (DBR) approach that applied theories of learning to evaluate the efficacy of design, instructional tools, or prototypes with students “in the wild” or ecologically valid settings (DBR Collective, 2003; Siek et al., 2014). The main objective of this methodology is to assess instructional tools in the ecologically valid real-world environment and to examine whether the tools positively influence students’ learning (Scott et al., 2020). From conducting an initial pre-assessment survey, we were able to identify that learners were particularly worried about their confidence in their abilities to learn online, motivations related to distance learning format, and whether or not learners would be able to critically engage with the course materials, as an overwhelming majority of students were first-time distance learners. After identifying the learning problems (Wang & Hannafin, 2005), we proceeded to develop solutions with digital learning tools through the application of LXD. Next, we evaluated the effectiveness of our learner experience course designs using evidence directly from students (Anderson & Shattuck, 2012). A longitudinal Pre-Post assessment design was used, in which outcome measurements are collected before and after the intervention (Craig et al., 2012). We selected the measurement method in which all student participants in this study underwent the intervention of the newly developed LXD based online learning environment. Analytically, we focused on the differences of outcomes for student measures from the same individuals between Time T1 (pre-intervention) and Time T2 (post-intervention) across 10 instructional weeks (White & Sabarwal, 2014) during the Spring 2020 academic term. By selecting this longitudinal research design method, we were able to control for temporal and secular changes in the outcomes observed (Leatherdale, 2019). Finally, we engaged in retrospective analysis for how our design outcomes were able to address our initial problems and further elucidated possible mechanisms to explain the theoretical underpinnings of LXD approaches (Fig. 2).

### Online learning experience design

The asynchronous self-paced online course focused on teaching Evolutionary Psychology through digital media and educational technologies. The online courses were hosted in Canvas, the university’s learning management system (LMS), and consisted of two randomly enrolled, identically sized, and closely scheduled classes in the School of Biological Sciences taught by the same professor over 10 instructional weeks. Cognizant of the research behind effective online learning environments, these online modules were designed to be flexible, interactive, and learner-centered (Floor, 2018; Hawley & Valli, 2000). The curriculum delivery incorporated an innovative self-paced learning experience and digital media features such as high-end studio production quality, 4 K multi-camera videos, green screen inserts, voice-over narrations, and animated infographics.

Emphasizing immersion and real-world applications, the online courses were developed within a situated cognition theory (SCT) for e-learning experience design (Brown et al., 1989). Operationalizing situated cognition theory, this online course design was grounded in practical elements of modeling, coaching, scaffolding, articulation, reflection, and exploration (Collins et al., 1991). More specifically, the 80-min long lessons were chunked into smaller three to five-minute scaffolded video phases instead of one long continuous stream to reduce fatigue, cognitive load, and opportunities for students to mind-wander (Mayer, 2019). These video scaffolds were designed to pre-train students in general concepts and terminologies with scientific visuals and simplified explanations, prior to engaging in the more in-depth and detailed study with a textbook reader. Subsequent to each video scaffold, the lecture questions that followed served as low-stakes content practice and retention exercises for learners to verify their accuracy while developing conceptual understanding. After this initial pretraining, students would engage in their readings of the text, followed by taking a quiz corresponding to the video lecture which assessed content mastery. Concurrently with, or following the quizzes (according to student choice), students were required to respond to journal reflection prompts, applying the concepts learned from the video scaffolds. Three types of journal reflection prompts were utilized in this course: perspective prompts, metacognitive prompts, and empirical prompts. Perspective prompts focus on assessing learners' understanding and application of the concepts of evolutionary psychology and give learners opportunities to synthesize new ideas based on the dynamics they learn about in this course. Metacognitive prompts challenge learners to analyze their developing ideas about evolutionary psychology, and what impact these ideas might have on their worldview and, in some cases, broader culture. Lastly, empirical prompts ask learners to try something and report back on their results. For example, in some cases, learners might be asked to discuss something with family or friends. Learners might even be asked to try practicing elements of ancestral human behavior or culture, gleaned from anthropology, to see if there are any experiential effects worth noting in their journals. A key element of successful LXD is the primary focus of designing for human-centered learning and human behaviors throughout the learning process (Floor, 2018). These LXD principles were applied by designing each asynchronous activity to be goal-oriented and learner-centered. Specifically, students experience completing many small scoring assignments that contribute to and culminate in applying concepts to questions about their own personal perspectives and contextual experiences about their own lives. Furthermore, these design choices enable students to actively engage in their own productive metacognitive judgments and motivations to reflect on “how and why” they arrived at their solutions, which has been found to foster learners’ critical thinking and use of elaboration skills within an online learning environment (Tullis & Benjamin, 2011; Wang et al., 2013a, 2013b).

Moreover, drawing on best practices of user experience UXD careful considerations were made within the course interface to promote student ease of use, findability, and navigability (Simunich et al., 2015). This was accomplished by implementing a novel interface design with a “dashboard-style” course introduction page to organize assignments, lecture videos, quizzes, and additional course materials. This served as a “course guide” to help students navigate their learning experience in a progressive manner. As a result, the online course was designed for students to enter the course space and ultimately land on the weekly “course guides” with all of the pertinent resources, assignments, and quizzes located centrally in one space. These asynchronous activities were provided to establish a systematic routine for learners to adopt throughout the 10 weeks of online instruction. This also served to promote flexibility and greater student autonomy within the course, as videos were available for students to play, pause, rewind, and fast forward with closed captioning for greater content accessibility. Efforts to support human–computer interactions through thoughtful UXD were also invested to reduce confusion and frustration (Shneiderman & Hochheiser, 2001), redirecting student efforts toward learning the content, rather than toward worrying about learning how to access the content in the LMS (Hu, 2008). Designing a system that is more usable and human-centered was sought to enhance learners' control and interaction with the information presented. Thus, the course’s design intentions were meant to ignite students' motivations and train students to adapt their learning behaviors such as their engagement, critical thinking, and elaboration within the asynchronous self-paced online course.

### Instrumentation

Data in this study was collected electronically. All of the measures employed were distributed to participating students and collected online via Qualtrics XM during the Spring 2020 academic term. Participants were provided a direct link to the surveys which were embedded within the Canvas LMS course space. Student online learning self-efficacy data was collected using the self-report Online Value and Self-Efficacy Scale (OLVSES) (Artino & McCoach, 2008). This instrument was developed by Artino and McCoach (2008) to measure students' self-efficacy and task-value for learning specifically within a self-paced, online course. The OLVSES instrument contains a total of 11 questions within two sub-constructs. Each question was scored on a 7-point Likert scale ranging from 1 (*completely disagree*) to 7 (*completely agree*). Artino and McCoach (2008) report the internal consistency coefficients (Cronbach alphas) for self-efficacy and task-value are 0.92 and 0.89, respectively.

The Motivated Strategies for Learning Questionnaire (MLSQ) instrument was developed by a team of researchers from the National Center for Research to Improve Postsecondary Teaching and Learning and the School of Education at the University of Michigan (Pintrich et al., 1993). The MLSQ is a self-report psychometric designed to assess undergraduate students’ motivations and their usage of varying learning strategies. Response options were designated on a 7-point scale, 1 (*completely disagree*) to 7 (*completely agree)*. The subscales of self-regulation, elaboration, and critical thinking were utilized in this study to evaluate students in a self-paced online course. Pintrich et al. (1993) report that the internal consistency coefficients (Cronbach alphas) are 0.79, 0.76, and 0.80, respectively.

Students’ perceived online engagement was measured using a 12-item survey (Rossing et al., 2012). Response options were designated on a 5-point scale, 1 (*completely disagree*) to 5 (*completely agree)*. The survey consisted of a combination of questions about students’ perceptions of learning and their perceived engagement in an online course. The internal consistency coefficient (Cronbach alpha) for this instrument is 0.90.

### Data analysis

#### Quantitative analysis

Descriptives and scale reliability checks were conducted to verify the alpha coefficients for all of the validated instruments used in this study (Table 2). All of the variables measured in this study were analyzed by first recoding the Likert questions into their respective positive or negative values, followed by computing the means of the items associated with each subscale. Paired sample t-tests were conducted to assess the change in students’ social cognitive motivational traits and learning behaviors at two-time points (pre and post) over the 10-week instructional period. Bivariate correlations evaluated the relationships between students’ motivational traits and learning behaviors (See Table 2). Lastly, multiple regression analyses were conducted to estimate the association of students’ social cognitive motivational traits (self-efficacy, task-value, self-regulation) as independent predictors for students’ learning behaviors (engagement, elaboration, critical thinking).

**Table 2:** Descriptive statistics and correlations for study variables

Variable	*n*	*M*	*SD*	α	1	2	3	4	5	6
1. Self-efficacy	181	5.92	0.719	0.903	–					
2. Task-value	181	5.63	0.871	0.919	0.425**	–				
3. Self-regulation	181	4.46	0.970	0.814	0.310**	0.368**	–			
4. Engagement	181	3.40	0.711	0.900	0.476**	0.380**	0.373**	–		
5. Elaboration	181	5.54	0.859	0.887	0.408**	0.659**	0.484**	0.350**	–	
6. Critical Thinking	181	5.16	0.975	0.859	0.357**	0.521**	0.476**	0.302**	0.584**	–

**Correlation is significant at the 0.01 level (2-tailed)

### Qualitative analysis

Qualitative analysis of student evaluation responses involved data analysis through Qualtrics Research Core XM text analysis software. A deductive coding approach, or concept-driven coding method (Saldaña, 2021), was selected for analyzing students’ post-assessment free-response questionnaire data in order to confirm the validity and reliability of our analytical findings. Through this process, analytic memos were written, while pre-defined subcodes and anchor codes were developed and systematically applied based on our quantitative variables (See Table 7). Inclusive and exclusive statements were clearly written to differentiate code applications. After reaching saliency, corroboration of quantitative and qualitative results further discerned potential mechanistic interactions. Researchers in this study made use of spot-checking in order to reach reliability and reduce bias throughout the coding process.

## Results

### Paired-sample T-tests

Paired-samples (2-tailed) t-tests were conducted to assess the changes in students’ social cognitive motivation variables (self-efficacy, task-value, self-regulation) and learning behaviors (engagement, elaboration, and critical thinking) throughout the 10-week instructional period. As Table 3 indicates, there was a statistically significant increase in students' self-efficacy from pretest (*M* = 5.39, *SD* = 0.87) to posttest (*M* = 5.90, *SD* = 0.70), *t*(161) = 7.48, *P* < 0.001. The mean increase of students’ self-efficacy throughout the 10-week quarter was 0.51 with a 95% confidence interval ranging from 0.38 to 0.65. The effect size for this analysis was medium (*d* = 0.59). The results from the pre-test (*M* = 5.47, *SD* = 0.77) and post-test (*M* = 5.67, *SD* = 0.84) of students’ task-value indicate a statistically significant increase throughout the quarter, resulting in a mean increase of 0.19 with a 95% confidence interval of 0.08 to 0.31, *t*(161) = 3.30, *P* < 0.001. The effect size for this analysis was medium (*d* = 0.59). There was a statistically significant increase in students' self-regulation from pretest (*M* = 4.31, *SD* = 0.89) to posttest (*M* = 4.48, *SD* = 0.97), *t*(161) = 2.38, *P* < 0.05. The mean increase of students’ self-regulation throughout the 10-week quarter was 0.163 with a 95% confidence interval ranging from 0.03 to 0.29. The effect size for this analysis was small (*d* = 0.27). Additionally, the results from the pre-test (*M* = 4.86, *SD* = 0.99) to post-test (*M* = 5.20, *SD* = 0.98) of students’ critical thinking indicate a statistically significant increase throughout the quarter, resulting in a mean increase of 0.338 with a 95% confidence interval of 0.18 to 0.49, *t*(161) = 4.32, *P* < 0.001. The effect size for this analysis was medium (*d* = 0.34). However, the relationship between students’ elaboration learning strategy from pre-test (*M* = 5.58, *SD* = 0.73) and post-test (*M* = 5.59, *SD* = 0.84) was not statistically significant *t*(161) = 0.081, *P* = 0.936. Overall, the results from the paired samples t-tests indicate that students’ self-efficacy, task-value, self-regulation, and critical thinking were significantly increased and distinguishable from pre to post assessment throughout the 10-week instructional period.

**Table 3:** Results of Paired-samples t-tests examining undergraduates’ motivations and learning strategies

Study variables	Pretest		Posttest		95% CI for Mean Difference		*t*	*P*	Cohen’s *d*
	*M*	*SD*	*M*	*SD*	Lower	Upper			
Self-efficacy	5.39	0.874	5.90	0.701	0.376	0.647	7.48	< 0.001	0.588
Task-value	5.47	0.773	5.67	0.839	0.078	0.312	3.30	0.001	0.587
Self-regulation	4.31	0.887	4.48	0.975	0.028	0.299	2.39	0.018	0.267
Elaboration	5.58	0.730	5.59	0.840	− 0.108	0.118	0.081	0.936	0.006
Critical Thinking	4.86	0.994	5.20	0.976	0.184	0.493	4.32	< 0.001	0.340

This table includes the results from the paired samples t-test (2-tailed)

*M* mean, *SD* standard deviation, *CI* confidence interval, *d* effect size

### Correlations

Exploratory Pearson correlations were documented in Table 2. Students’ self-efficacy was positively related to students’ self-regulation (*r* = 0.310, *n* = 181, *P* < 0.01), usage of elaboration (*r* = 0.408, *n* = 181, *P* < 0.01), and critical thinking (*r* = 0.357, *n* = 181, *P* < 0.01). Meanwhile, students’ task-value was significantly associated with self-regulation (*r* = 0.368, *n* = 181, *P* < 0.01), engagement (*r* = 0.380, *n* = 181, *P* < 0.01), elaboration (r = 0.659, *n* = 181, *P* < 0.001), and critical thinking (*r* = 0.521, *n* = 181, *P* < 0.01). Furthermore, students’ self-regulation was significantly correlated with engagement (*r* = 0.373, *n* = 161, *P* < 0.01), elaboration (*r* = 0.484, *n* = 181, *P* < 0.01) and critical thinking (*r* = 0.476, *n* = 181, *P* < 0.01).

### Multiple regression analyses

It has been hypothesized by learning experience designers that the underlying mechanisms underpinning LXD and students’ learning behaviors are likely to be the result of increasing social cognitive motivational factors. To determine the association between student social cognitive motivation variables and learning strategies, multiple regression analyses were conducted. Three independent variables (self-efficacy, task-value, and self-regulation) of social cognitive motivation factors were used to predict the dependent variables (engagement, elaboration, and critical thinking) of learning strategies. Student socioeconomic characteristic variables such as gender, low income, underrepresented minority, and first-generation were analyzed during preliminary analysis. None of these student variables were significantly related to the outcome variables. As such, these student characteristic variables were not retained in the final regression models. Table 4 provides a detailed summary of the regression analyses for each of the predictors on the outcome variables.

**Table 4:** Multiple regression analysis predicting online engagement from motivational variables

Measure	Engagement			
	*R*^2^	*B*	*SE B*	*ß*
Step 1	0.220			
(Constant)		1.47	0.274	
Self-efficacy		0.367	0.052	0.469***
Step 2	0.286			
(Constant)		0.390	0.347	
Self-efficacy		0.307	0.052	0.392***
Task-value		0.217	0.054	0.267***
Step 3	0.312			
(Constant)		0.390	0.347	
Self-efficacy		0.278	0.052	0.355***
Task-value		0.172	0.056	0.212**
Self-regulation		0.131	0.050	0.179**

*P < 0.05. **P < 0.01. ***P < 0.001. *ß*, standardized coefficient. *B*, unstandardized coefficient. *SE B*, standard error

In Model 1, we assessed the relationship between students’ self-efficacy, task-value, and self-regulation on students’ online engagement (See Table 4). At step 1, students’ self-efficacy (*β* = 0.469, *P* < 0.001) was significantly predictive of their online engagement. In step 2, the addition of task-value (*β* = 0.267, *P* < 0.001) was statistically significant, accounting for an additional 6.6% of the explained variance (See Table 4). In step 3, the association between students’ self-regulation and online engagement was statistically significant (*β* = 0.179 *P* < 0.01). This final inclusion of self-regulation explained an additional 2.7% of the variance *R*^2^ = 0.312, *F*(3, 178) = 26.7, *P* < 0.001 (See Table 4). On average, these results indicate that students’ self-efficacy, task-value, and self-regulation within the online learning environment were significantly predictive of their online engagement, accounting for 31.2% of the explained variance.

Model 2 estimated the association of students’ motivational traits and self-regulation on their elaboration learning strategy while controlling for their elaboration pretest scores (See Table 5). Results indicate that self-efficacy (*β* = 0.460, *P* < 0.01) was significantly predictive of students’ elaboration learning strategy at step 2. In step 3, the addition of task-value was statistically significant, accounting for a 16.7% increase in the explained variance. In the final step, the association of self-efficacy (*β* = 0.133, *P* < 0.05), task-value (*β* = 0.414, *P* < 0.001), and self-regulation (*β* = 0.203, *P* < 0.01) were all significantly predictive of students’ elaboration. This accounted for 57% of the explained variance *R*^*2*^ = 0.570, in the model *F*(3, 177) = 50.9, *P* < 0.001 (See Table 5).

**Table 5:** Multiple regression analysis predicting students’ elaboration from motivational variables

Measure	Elaboration (Post)			
	*R*^2^	*B*	*SE B*	*ß*
Step 1	0.314			
(Constant)		2.02	0.425	
Elaboration (Pre)		0.076	0.076	0.561***
Step 2	0.370			
(Constant)		1.39	0.442	
Elaboration (Pre)		0.526	0.079	0.460***
Self-efficacy		0.241	0.065	0.256***
Step 3	0.537			
(Constant)		0.318	0.406	
Elaboration (Pre)		0.327	0.073	0.286***
Self-efficacy		0.163	0.057	0.173**
Task-value		0.458	0.061	0.464***
Step 4	0.570			
(Constant)		0.193	0.395	
Elaboration (Pre)		0.297	0.071	0.260***
Self-efficacy		0.126	0.056	0.133*
Task-value		0.408	0.061	0.414***
Self-regulation		0.172	0.050	0.203**

*P < 0.05. **P < 0.01. ***P < 0.001. *ß*, standardized coefficient. *B*, unstandardized coefficient. *SE B*, standard error

In Model 3, we estimated the association of students’ social cognitive motivational traits on their critical thinking learning behaviors by conducting multiple regression analyses while controlling for students’ critical thinking pretest scores (See Table 6). At step 2, self-efficacy (*β* = 0.194, *P* < 0.01) was significantly predictive of students’ critical thinking. In step 3, the inclusion of task-value was statistically significant, accounting for a 10.2% increase of the explained variance. In the final step, the students’ self-regulation (*β* = 0.252, *P* < 0.001) was significantly predictive of their critical thinking, accounting for an additional 5.0% of the explained variance. These results indicate that students’ online self-efficacy, task-value, and self-regulation were significantly predictive of students’ critical thinking in the online course *R*^2^ = 0.471, *F*(3, 178) = 34.3, *P* < 0.001.

**Table 6:** Multiple regression analysis predicting students critical thinking from motivational variables

Measure	Critical Thinking (Post)			
	*R*^2^	*B*	*SE B*	*ß*
Step 1	0.251			
(Constant)		2.82	0.333	
Critical thinking (Pre)		0.097	0.013	0.501***
Step 2	0.319			
(Constant)		1.55	0.451	
Critical thinking (Pre)		0.428	0.066	0.441***
Self-efficacy		0.297	0.075	0.268**
Step 3	0.421			
(Constant)		0.232	0.488	
Critical thinking (Pre)		0.067	0.013	0.344***
Self-efficacy		0.194	0.072	0.175**
Task-value		0.407	0.078	0.352***
Step 4	0.471			
(Constant)		0.029	0.470	
Critical thinking (Pre)		0.058	0.012	0.298***
Self-efficacy		0.135	0.071	0.122*
Task-value		0.337	0.077	0.291***
Self-regulation		0.252	0.066	0.253***

*P < 0.05. **P < 0.01. ***P < 0.001. *ß*, standardized coefficient. *B*, unstandardized coefficient. *SE B*, standard error

In summary, students’ social cognitive motivational traits were positive and significantly different between Time T_1_ (pre-intervention) and Time T_2_ (post-intervention) across 10 instructional weeks. In addition, students’ social cognitive motivational traits were significantly predictive of students' learning behaviors. As a result, as students’ self-efficacy, task-value beliefs, and self-regulation factors increased while participating in this LXD based online course, on average, their engagement, usage of elaboration, and critical thinking skills increased. Interestingly, the stepwise addition of students’ task-value beliefs as a predictor increased the proportion of the explained variance in each model significantly, above and beyond self-efficacy and self-regulation. For models 1, 2, and 3, the increase in explained variance after the addition of students’ task-value beliefs was 6.6%, 16.7%, and 10.2% respectively.

### Students’ learning experience within the online course

To obtain a more nuanced understanding of students’ learning experience within the online learning environment, we analyzed students’ official anonymized course evaluations and free-response data from the post-assessment questionnaire (See Table 7). We took a qualitative approach to analyze students’ learning experiences during their participation within the online course to further triangulate our qualitative and quantitative findings. Analysis of the qualitative data from students’ questionnaire responses provided evidence regarding how the course user interface and UXD supported students’ experience within the LX-designed online learning environment. Three key patterns about the students’ learning experience emerged – findability, video navigability, and self-pacing. Representative samples of students’ descriptions and perspectives are provided on each theme.

**Table 7:** Codebook used for the qualitative content analysis of learners' open-ended survey responses

Codebook of student evaluation responses
Unit of analysis: Student course evaluations and post-assessment questionnaire responses:
1. Please elaborate on what aspects of the online format might have helped your learning experience in this online course
2. What are the strengths of the online course learning experience?
3. What are the weaknesses of the online course learning experience?
4. How can the technology for this course be improved to support your learning experience?

Codes	Definitions	Example
*Analytic Category: Findability*		
Deadlines	Findability was defined as references to the quick identification of course materials, course structure and organization, and content accessibility	*Student A*: “The way the course was set up, from the online lectures to the quizzes, all helped me better organize my time allocated for this course.”
Structure	*Inclusion: structure, searching, course organization, layouts, content, flow, scaffolding, content accessibility, ease of use*	*Student B*: “The accessibility to everything at any time. Easy to navigate, I really enjoyed the lecture videos and how organized they were.”
Organization		*Student C*: “For this online course specifically, it was very organized and straightforward. This contributed to my success in this class.”
Dashboard	*Exclusion: exclude if mentioned in the context of navigation, video playback*	*Student D*: “Due dates always show up on the Canvas dashboard, which serves as an online agenda for me. Instructions for assignments are always there for me to look back on.”
		*Student E*: “I think that the aspects of this course that helped me are all the videos and how this course was formulated for an online class and it was easy and accessible. This made it easier to watch it on my phone and easier to access than zoom recorded lectures.”
*Analytic Category: Video Navigability*		
Video Playback	We defined video navigability as references to the video user interface and the learner’s ability to manipulate the video playback options. Such playback options include play, pause, fast forward, rewind, speed up, slow down, toggle full-screen, toggle closed captions, and enable transcripts	*Student F*: “A strength of the online course is that I can complete online assignments any time I want. Another strength is that video lectures can be slowed down or replayed for me to take notes or if I did not understand a part of the lesson.”
Play	Play: the user action to start to a video	*Student G*: “The ability to pause and go back in lecture videos was very helpful in helping me understand difficult concepts that I had to keep going back to in order to fully comprehend.”
Pause	Pause: the user action of stopping a video	*Student H*: “I learn better by videos and then in person, because we have the ability to rewind, slow, down, speed up, increase volume. If I happen to miss it in class it can be difficult to catch up.”
Speed-up	Speed-up: the user action to increase the speed of the video	*Student I*: “I have the freedom to watch and rewatch lecture videos when I have time, in order to better understand the content. It is a lot easier to take notes since I can pause the videos whenever and take a moment to understand what I am writing before the lecture moves on.”
Slow-down	Slow-down: the user action to decrease the speed of the video	*Student H*: “Having recorded lectures allows for students to play back the video and take it at their own speed, whereas in person lectures might not all offer recorded lectures. It has allowed me to learn new, better study habits.”
Rewind	Rewind: the user action to go back to a previous timecode of the video	
Fast-forward	Speed-up: the user action to go forward to a future timecode of the video	
Maximize	Maximize: the user action to increase the size of the video player	
Minimize	Minimize: the user action to decrease the size of the video player	
Captions	Captions: the user action to enable subtitles as the video plays	
	*Inclusion: references to video playback options, course navigation, speed, video interface, flexibility, learner-choice for accessing content*	
	*Exclusion: exclude if mentioned in the context of course structure, organization, scaffolding*	
*Analytic Category: Self-pacing*		
Video Playback	Self-paced learning was defined as references to autonomy, on your own time, and time frames with regards to pacing while participating in the online course such that students could easily navigate the course space freely to re-watch, pause, play a video, complete assignments on their own time, and access the course at their own leisure	*Student I*: “One of the strengths of online learning is that we get to go at our own pace. If we have a lot of assignments we need to time ourselves so that we get things done according to what is best for us.”
Speed	Speed: the specific pacing it takes to complete a lesson, relative speed of completing videos, assignments, and quizzes in relation to others	*Student J*: “Online format gave me the chance to study the material on my own time; I wouldn't have had time to truly learn the material if it was during the official time indicated. It was really interesting, and I wouldn't have enjoyed it as much if it was in a traditional setting”
Flexibility	Flexibility: to complete on your own time, freedom to learn when it suits the learner, school from home, location can be anywhere	*Student K*: “I was able to do everything on my own time. I succeed when I don't feel like I am pressured to complete something specifically on that day. The format where assignments/lectures are given early to complete helps me stay on track.”
Schedule	Schedule: mentioning convenience of one’s own schedule, non restrictive, convenience to set own schedule and plans	*Student L*: “The pacing can teach students to overcome obstacles, problem solve, find creative solutions to problems, manage their time better, and improve study habits. In addition, the pace made the journals interesting and fulfilling to answer. The journal entries caused me to think in depth about evolutionary psychology and apply it to my own life”
	*Inclusion: include if in reference to freedom to complete course on your own time, location, setting, time management, autonomy*	*Student M*: “This self-paced online learning has allowed me to learn new, better study habits. I have been better about staying on top of the material and learning and finding solutions to problems on my own. We also got to talk about our own opinions based on what we read and watched in videos for our journals to demonstrate comprehension. It made the class interesting.”
	*Exclusion: don’t include if simply describing accessibility through devices, video navigation, or course organization*	

#### Findability

Findability was defined as references to the quick identification of course materials, course structure and organization, and content accessibility (See Table 7). As a UXD decision, the course dashboard was employed for the intent of increasing usability so that students might find it easier to locate all of the week’s materials such as videos, PDFs, quiz links, and additional supporting resources, having them located centrally all in one course page. It was hypothesized that this dashboard design would serve to provide direct access to the course content to students in a quick and consistent manner, indicating due dates, course objectives, and learning goals to effectively increase findability. *Student A:* “The way the course was set up, from the online lectures to the quizzes, all helped me better organize my time allocated for this course.”

- *Student B*: “The accessibility to everything at any time. Easy to navigate, I really enjoyed the lecture videos and how organized they were.”
- *Student C*: “For this online course specifically, it was very organized and straightforward. This contributed to my success in this class.”
- *Student D*: “Due dates always show up on the Canvas dashboard, which serves as an online agenda for me. Instructions for assignments are always there for me to look back on.”
- *Student E*: “I think that the aspects of this course that helped me are all the videos and how this course was formulated for an online class and it was easy and accessible. This made it easier to watch it on my phone and easier to access than zoom recorded lectures.”

#### Video navigability

We defined video navigability as references to the video user interface and the learner’s ability to manipulate the video playback options (See Table 7). Such playback options include play, pause, fast forward, rewind, speed up, slow down, toggle full-screen, toggle closed captions, and enable transcripts. As a UXD decision, every video that was produced for this online course was published and embedded within the Canvas LMS with all of these playback options in mind. Our design intentions were to enable flexibility and learner-centered navigation options to provide opportunities for students to re-watch, pause, and play a video if they did not fully grasp the concepts during their first time through.

- *Student F*: “A strength of the online course is that I can complete online assignments any time I want. Another strength is that video lectures can be slowed down or replayed for me to take notes or if I did not understand a part of the lesson.”
- *Student G*: “The ability to pause and go back in lecture videos was very helpful in helping me understand difficult concepts that I had to keep going back to in order to fully comprehend.”
- *Student H*: “I learn better by videos and then in person, because we have the ability to rewind, slow down, speed up, increase volume. If I happen to miss it in class, it can be difficult to catch up.”
- *Student I*: “I have the freedom to watch and rewatch lecture videos when I have time, in order to better understand the content. It is a lot easier to take notes since I can pause the videos whenever and take a moment to understand what I am writing before the lecture moves on.”
- *Student H*: “Having recorded lectures allows for students to play back the video and take it at their own speed, whereas in person lectures might not all offer recorded lectures. It has allowed me to learn new, better study habits.”

#### Self-pacing

Self-paced learning was defined as references to autonomy, on your own time, and time frames with regards to pacing while participating in the online course such that students could easily navigate the course space freely to re-watch, pause, and play a video, complete assignments on their own time, and access the course at their own leisure (See Table 7). It was hypothesized that by providing clear instructions, usability descriptions, deadlines, and all of the week’s material in one space, while learners were particularly able to freely choose how to plan, monitor, and adjust their own study habits and schedules, that students’ self-efficacy, task-value, and self-regulation would positively impact their learning behaviors in the online course.

- *Student I*: “One of the strengths of online learning is that we get to go at our own pace. If we have a lot of assignments, we need to time ourselves so that we get things done according to what is best for us.”
- *Student J*: “Online format gave me the chance to study the material on my own time; I wouldn't have had time to truly learn the material if it was during the official time indicated. It was really interesting, and I wouldn't have enjoyed it as much if it was in a traditional setting”
- *Student K*: “I was able to do everything on my own time. I succeed when I don't feel like I am pressured to complete something specifically on that day. The format where assignments/lectures are given early to complete helps me stay on track.”
- *Student L*: “The pacing can teach students to overcome obstacles, problem solve, find creative solutions to problems, manage their time better, and improve study habits. In addition, the pace made the journals interesting and fulfilling to answer. The journal entries caused me to think in depth about evolutionary psychology and apply it to my own life”
- *Student M*: “This self-paced online learning has allowed me to learn new, better study habits. I have been better about staying on top of the material and learning and finding solutions to problems on my own. We also got to talk about our own opinions based on what we read and watched in videos for our journals to demonstrate comprehension. It made the class interesting.”

These commentaries provide additional measures of verification that the LXD approaches employed in this asynchronous online course aligned with the intended learner experiences. This resulted in students reporting sensitivities to the course being relatively easier to navigate, with course materials that were findable, and a course structure that directly supported their time management (See Table 7). The previous traditional in-person synchronous iteration of this same course did not contain these design features. Upon gleaning students’ official evaluations of that previous traditional course variant, comments of findability, navigability, or time management were not reported and may be inferred to potentially be uniquely specific to the intentional LXD approaches of this asynchronous course version. Likewise, perhaps the predominant distance-learning method typically employed by most instructors was a Zoom-mediated synchronous delivery that largely sought to more closely resemble traditional in-person methods, which are unlikely to contain the LXD approaches found in this investigation. Here, we note that while fully immersed in this online context, student subjects of the study were enrolled in all of their coursework online, with the preponderance of their courses occurring in Zoom-based synchronous approaches, while they experienced this experimental asynchronous LXD approach, providing realistic comparative sensitivity to the real-world efficacies of the LXD methods.

Further analysis of students’ comments on learning behaviors provided descriptive insights into the design advantages of the video interface. With students’ recounting of how the video playback choices,we might conjecture that student’s playback choices fostered more flexible and self-paced learning strategy behaviors that led to increased engagement with the course videos, elaboration through self-paced note taking and synthesis of new information, or perhaps contributed to capacities for critically thinking through difficult concepts. We also make note of students highlighting the affordances of video navigability in an online space that would otherwise not be possible in a traditional in-person face-to-face setting. Moreover, we observe instances of how the self-pacing nature of the course may be fostering new opportunities for students to develop confidence in their abilities to learn online, perceive aspects of online learning to be useful, and adapt their learning behaviors for their own learning benefits. As a result, these student excerpts provided illuminating perspectives on how the design decisions and intentions of the course through a LXD lens may have positively influenced the association between students’ motivations and their online learning behaviors.

## Discussion

### Learning experience design

This design-based research (DBR) study, made possible through the rapid transition to online learning, fostered the examination, synthesis, and application of learning theories, investigating the potential advantages of learning experience design (LXD). Our LXD approach was intended to be reliable, comprehensible, and above all, usable (Shneiderman & Hochheiser, 2001) to broadly serve students’ needs and changing learning behaviors as identified in the pre-assessment. This study operationalized LXD by grounding the asynchronous self-paced online course with Situated Cognition Theory (SCT) as its pedagogical framework and then deploying user design heuristics to support learners’ user experience. The LXD approach aimed to address student concerns through an empathy approach that was human-centered, goal-oriented, interdisciplinary, based upon theories of learning and practice to support students’ online learning experience. This study concurrently tracked LXD approaches with resultant student learning behaviors to examine an array of emergent factors to inform future digital teaching and learning design decisions. Few studies have explored highly autonomous self-paced online STEM courses grounded in LXD in situ*,* at an R1 university setting. In this study, LXD techniques were rapidly deployed to expedite measurements illustrating how the combination of user experience and learning design has the potential to provide learner-centered affordances to support undergraduate STEM students. When online learning becomes a dominant model of higher education, methods including asynchronous, synchronous, and blended approaches may benefit from investigations highlighting the affordances and constraints of LXD made during the pandemic time of fully online learning immersion to better prepare instructors, researchers, and designers in how to directly supports students’ social cognitive motivational traits and learning behaviors.

### User experience design

The qualitative analysis, which was discharged to complement our quantitative analyses, characterized our understanding of the course’s usability for learners with respect to user experience design (UXD). As design decisions, we developed a weekly “course dashboard” displaying all of the pertinent videos, assignments, and quizzes organized centrally in one space to serve as a course roadmap to make content easy to find. In addition, we promoted student flexibility by ensuring a wide variety of learner-centered video playback choices for students to play, pause, rewind, fast forward, and toggle closed captioning for greater student autonomy, content navigability, and ease of use. Upon analyzing student commentaries, we documented evidence suggesting that these usability design facets led to increased learner ease of use, findability, and video navigability of course materials. This, in turn, fostered a self-paced learning environment for students to develop, plan, monitor, and adjust their own study schedules and learning behaviors. More specifically, we recorded instances of students specifically describing ways that usability manipulations directly influenced how they engaged with the course materials, synthesized new information, and thought critically on their reflective metacognitive journal assignments (See Table 7). The results of this study are in line with previous research that has identified how strategic manipulations to the course usability promote quality user experience design by facilitating findability and navigability (Simunich et al., 2015). Efforts to support human–computer interactions (HCI) by drawing on UXD heuristics reduce confusion and frustration of locating course materials (Shneiderman & Hochheiser, 2001), redirecting student efforts toward learning the content, rather than toward worrying about learning how to access the content in the LMS (Hu, 2008). Such findings are consistent and suggestive that the usability considerations applied in this LXD based course promoted students’ user experience.

### Learning design pedagogy features

**Fig. 3:**
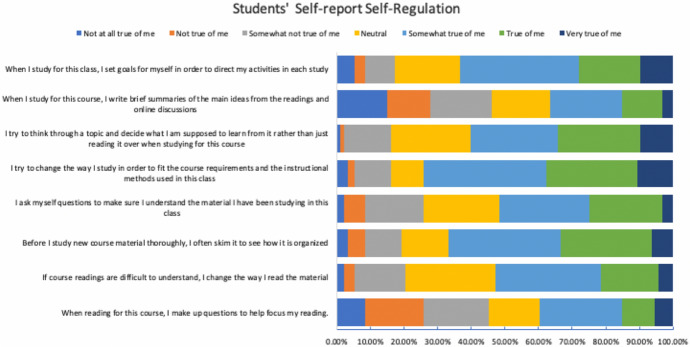
Students’ self-reported self-regulation at the end of the academic quarter. Response values were normalized to percentages and stacked horizontally for visual representations

**Fig. 4:**
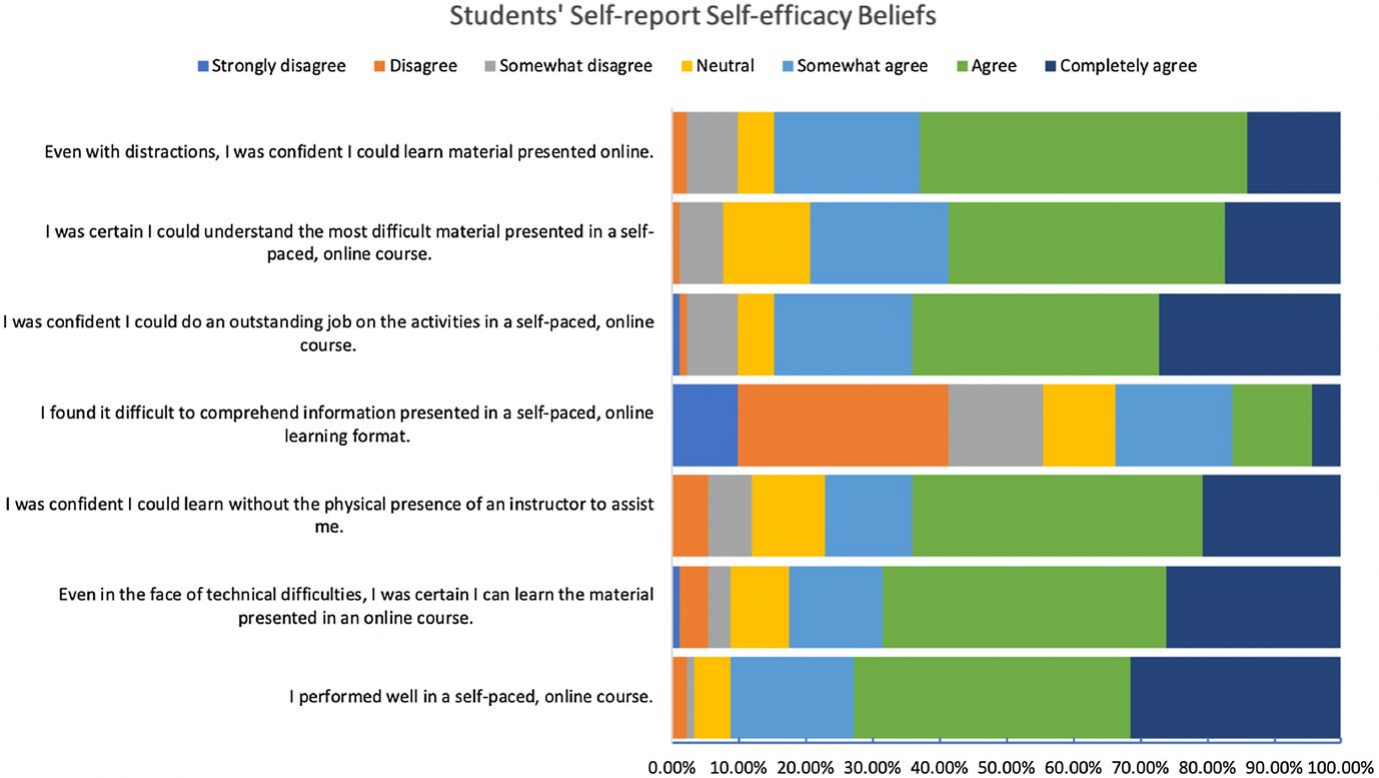
Students’ self-reported self-efficacy at the end of the academic quarter. Response values were normalized to percentages and stacked horizontally for visual representations

The online course was developed within a SCT framework for e-learning experience design to emphasize “learning by doing” (Brown et al., 1989). This pedagogical learning design method was chosen specifically to ground the learning experience in practical elements of modeling, coaching, scaffolding, articulation, reflection, and exploration (Collins et al., 1991). For example, to reduce learner disengagement through lack of instructor presence (Sorensen et al., 2017), we overlaid the instructor’s camera feed as a picture in picture within the main content video stream. Additionally, the scaffolded video experiences were designed to train students to systematically navigate their learning experience within the course: the videos served to pre-train students in general concepts and terminologies with scientific visuals and simplified explanations; lecture questions afforded opportunities for rehearsal and practice; the course reader provided conceptual understanding; and the metacognitive reflective journals guided learners toward conceptual applications. We observed that 74.2% of students found that they tried to change the way they studied in order to fit the course requirements and the instructional methods used in the course (See Fig. 3). 77.2% of students were confident that they could learn without the physical presence of the instructor to assist them (See Fig. 4). Moreover, 86.1% of students found that the course materials in this self-paced online course were useful for their learning experience (See Fig. 4). Such findings begin to enhance our understanding of the effectiveness of the SCT instructional pedagogy grounding our LXD approaches.

### Linking LXD, motivation, and learning strategy behavioral outcomes

The underlying mechanisms underpinning LXD impact on students’ learning behaviors are hypothesized to be the catalyzing result of dynamically increasing social cognitive motivational factors. When quantitatively measuring students' change in their social cognitive motivation variables throughout the 10-week instructional period, the mean differences between students’ self-efficacy, task-value beliefs, and self-regulation were positive, significantly different, with a medium-sized effect (See Table 3). We might attribute these positive increases as a direct result of our LXD applications, specifically the combined impacts of grounding the online course in learning design pedagogy and user experience design. Furthermore, when multiple regressions were conducted to further explain students’ social cognitive motivational impacts on their learning behaviors, the results revealed significant predictions on students’ engagement, elaboration, and critical thinking skills. The subsequent paragraphs below detail the resulting impacts of how students' social cognitive motivational factors influenced their learning strategy behaviors.

### Engagement

Results revealed that students’ self-efficacy, task-value, and self-regulation significantly predicted students’ online engagement (See Table 4). Specifically, students with higher levels of self-efficacy (*β* = 0.355, *P* < 0.001), task-value (*β* = 0.212, *P* < 0.01), and self-regulation (*β* = 0.179, *P* < 0.01), on average, demonstrated higher levels of online engagement, suggesting that it is important to target students’ social cognitive motivational factors in order to facilitate students’ engagement within an online course (See Table 4). Several key LXD facets may explain this positive trend in students’ engagement. As evidenced by the representative sample of student commentaries, the usability and user interface of the online course promoted course structure, organization, ease of use, and findability. Such elements in UXD offer affordances to learners to not only develop confidence in their abilities to access the course materials, but also perceive that the course materials are valuable in a way that supports their learning needs. In addition, the SCT video scaffolded pedagogical framework was designed to train students in how to systematically operate the course, providing an instructional protocol for distance learners to plan, monitor, and adopt, thereby facilitating their engagement with the course materials. When comparing all three independent predictors, we found that students’ task-value beliefs contributed significantly more toward students’ engagement, above and beyond self-efficacy and self-regulation. We suspect that this may be the case because our LXD approaches are contributing toward students elucidating the theorized benefits of asynchronous self-paced online learning.

### Elaboration

Students’ self-efficacy (*β* = 0.133, *P* < 0.05), task-value (*β* = 0.414, *P* < 0.001), and self-regulation (*β* = 0.203, *P* < 0.01) significantly predicted students’ use of elaboration (See Table 5). On average, these findings suggest that as students’ social cognitive motivational factors increased, so did their elaboration learning behaviors. Interestingly, among self-efficacy, task-value, and self-regulation, students’ task-value beliefs were recorded (see Table 2) to have the largest correlation coefficient value when observing the associations of students’ social cognitive variables with their use of elaboration. This pattern continues to persist when observing the multiple regression analyses. The addition of task-value as a predictor in the stepwise blocks revealed the largest r-square increase, above and beyond self-efficacy and self-regulation (See Table 5). This might be explained by the comments from students highlighting how the video user interface design afforded navigability options for students to play, pause, rewind, fast forward, and read closed captioning that would ordinarily not be possible in an in-person traditional lecture hall. Additionally, students have also commented on how the video playback options allow adaptive note taking, with the ability to pause the videos at their own leisure to take a moment and understand what they were writing. While our initial hypothesis indicated that self-regulation would contribute more significantly, these findings, however, are consistent with Artino and McCoach (2008), indicating that students’ task-value may be more important than self-efficacy and self-regulation when considering highly autonomous and flexible self-paced online courses.

### Critical thinking

Analysis revealed that students’ self-efficacy (*β* = 0.122, *P* < 0.05), task-value (*β* = 0.291, *P* < 0.001), and self-regulation (*β* = 0.253, *P* < 0.001) were significantly predictive of students’ critical thinking skills, suggesting that on average, as students’ social cognitive motivational variables increased, so did their critical thinking skills (see Table 6). A similar trend was noted with students' task-value beliefs, with task-value explaining an additional 10.2% of the variance (See Table 6). Several key learning design features may explain this trend. By chunking the videos into bite-sized segments and scaffolding them by coherent conceptual topics, the course structure was designed to reduce cognitive load, lower frustration, and promote the accommodation of new conceptual information (Meyer, 2019). In addition, rather than administering midterms or final assessments, as a curriculum design decision, the choice was made for students to apply their critical thinking skills by submitting metacognitive conceptual journals. These conceptual journals challenged learners to analyze their developing ideas about evolutionary psychology, what impact these ideas might have on their worldview, and in some cases, broader culture, assessing understanding and real-world application of the concepts. When gleaning from student excerpts, we documented instances of students perceiving that the pacing of the course taught them to overcome obstacles, problem-solve, and find creative solutions to improve their study habits (See Table 7). Moreover, students noted how the pacing of the course made the journal prompts fulfilling to answer. Attuning our understanding of the LXD impacts through the integration of learning design principles with learner-centered user experience heuristics may have helped to discern the resulting increase of students’ critical thinking skills as explained by their developing motivations within the course.

### Limitations

Future research is warranted to further examine the limitations to and affordances for undergraduate science online teaching and learning that may be gained for future designs. This design-based research (DBR) study was the first iteration of a multi-year LX-design-based in situ implementation project. Future research studies would benefit from the experimental manipulations of LXD based courses to further develop, validate, and scale the efficacies of asynchronous online learning environments. In addition, as students continue to grow in their learning experience with different formats of online learning, including synchronous, asynchronous, and hybrid models, such DBR studies would be able to discern potential causal mechanistic relationships between various learning formats and detect which are better suited for undergraduate STEM courses at the R1 instructional level. Another limitation in our study was that all of the measures utilized were self-reported. Self-report data inherently have biases as they serve as an interpretation of the students' perception. However, with increasing access to learning analytics data gained through the improving Canvas LMS, in future studies, we will be exploring students’ retrospective course log data such as page views, participation, time-on-task, and completion rate to further corroborate student interactions and behaviors in online learning environments.

### Constraints, affordances, and implications for practice

As a part of our evaluation phase within the DBR approach, design constraints for student learners were noted. For example, large quantities of segmented videos can become difficult to keep track of for students who fall behind in the online course; some students may prefer features for tracking the videos based on completion; the lack of direct face to face interactions for students to immediately raise questions with the instructor or socialize with their peers in the course may be a downside for many; although students could communicate via email to discuss difficult concepts, reaching out to the professor this way could present an added barrier. Through this retrospective analysis to critically evaluate our design, we have begun to identify ways in which we can iterate our current designs to focus on these unintended design constraints. This includes adding a system progress indicator bar, system interface feedback (i.e. checkmarks, status indicators, analytics), tracking completed videos and assignments, and adding a FAQ section in between videos of aggregated questions. Further options to add a dynamically updating peer contributed message board embedded alongside videos with digital tools such as Padlet, are currently being explored. As a result, the reflective process guided by the DBR approach has allowed for student contributed perspectives in order to facilitate the next cycle of iterations in our LXD research.

For learning experience designers, the challenge is not only to develop learning environments that increase conceptual understanding by drawing on theories of learning sciences, but also to create learning experiences that are interesting, engaging, and support human-centered behaviors (Ahn et al., 2019). By designing with these principles in mind, we are better able to ensure that our LXD approaches specifically target the intended audience who stand to benefit the most, providing learning affordances to distance learners. Specifically, learners were particularly worried about their social cognitive motivational traits and learning behaviors, as identified in the pre-assessment. To directly address students’ needs, we designed an online course intervention for the purpose of increasing students' social cognitive motivations (self-efficacy, task-value, self-regulation) and learning behaviors (engagement, elaboration, critical thinking) by grounding the online learning environment in practical elements of learning and user experience design.

From a pedagogical learning design perspective, the LXD choices to create a video scaffolded learning experience that sequentially pre-trained students with immediate recall and retention questions, prior to reading the textbook, and ultimately requiring students to apply their conceptual knowledge in reflective journals, afforded students a systematic learning routine in an online space. As evidenced in our study, this established routine patterns and operational norms, which contributed to students' judgments about their confidence, perceived usefulness, and self-regulation, led to positive changes in their learning behaviors. These affordances dynamically increased students’ social cognitive motivational traits which led to positive impacts in learners’ online engagement, elaboration, and critical thinking. While more research is certainly warranted, we recommend that instructors and designers of STEM online courses in higher education adopt similar digital media tools (i.e. video production, segmentation, real-world connections) and pedagogical learning designs like the SCT framework (scaffolding, goal-setting, reflecting), providing explicit cues which give learners a means to adopt effective online learning strategies. We also argue that the affordances of the LXD approach were only made possible through the strategic simultaneous combination of learning and user experience design.

With careful considerations to user experience design, the LXD choices to incorporate usability manipulations that resulted in the creation of a course dashboard with centralized course content and video navigation autonomy, enabled design affordances that led to direct changes in learning behavior. The inclusion of learning dashboards, as a UXD based approach within an online learning environment, enables student learning processes which include sensemaking, awareness, reflection, and data processing (Ahn et al., 2019; Jivet et al., 2017). From establishing course structure to increasing findability, the design afforded students a single entry point within the course each week to identify, access, and plan for the required assignments and tasks. Moreover, the ability for learners to play, pause, rewind, and enable closed captioning on the videos afforded precise control and interaction for students to engage, elaborate, and critically interact with the course content. Such interactions enabled learners to pause and reflect on concepts or engage in summative and generative note taking for higher-order thinking. As such, we argue that the parallel affordances provided by bridging the literature on pedagogical learning and user experience design operationalize the benefits of STEM online courses grounded in learning experience design.

Through this DBR study, we have demonstrated how the affordances of LXD specifically influenced positive changes in student learning behaviors. Data from this study suggest that it is students' intuitions about their own confidence, value beliefs, and self-regulated skills that may be the driving factor linking LXD and the significant positive effects in student learning behaviors exemplified in this online course.

Recognizing students’ social cognitive motivational traits while learning remotely may further support students’ learning behaviors and the improvement of future iterations of online course delivery (Artino & McCoach, 2008; Eccles & Wigfield, 2002; Pintrich, 1999). We have also identified how our unintended design constraints presented new opportunities for refinement and iterations for the continual improvement of STEM online courses. Consequently, future online courses may benefit from this research and it may inform institutions on how to iteratively design and effectively foster successful online teaching and learning with the use of innovative learning experience design approaches over the more gradual transitions to modernized methods of digital learning of STEM courses in higher education.

## Conclusion

In summary, students’ social cognitive motivational traits increased significantly throughout the 10-week instructional term. Students’ self-efficacy, task-value, and self-regulation were also predictive of students’ engagement, usage of elaboration, and critical thinking. As a result, this study suggests that implementing asynchronous self-paced online courses with LXD approaches may positively impact students’ learning behaviors, potentially through influencing student’s social cognitive motivational traits. Results also suggest that students’ task-value beliefs may be most critical in explaining students’ learning behaviors when grounding online courses with LXD, above and beyond self-efficacy and self-regulation. Through our mixed-method approach, we further validated our quantitative analyses by qualitatively drawing on the rich descriptions of student’s learning experiences and the resulting impacts on their learning experiences. In particular, these descriptions explicitly pointed to the LXD features explaining how our design efforts contributed to students' motivations and changing learning behaviors in the course. Based on these collective findings, we recommend an instructor-designer DBR collaborative workflow to produce and design online courses with LXD approaches through the combination of pedagogical learning design and learner-centered user experience design considerations. This research study makes an important contribution to the field of STEM online teaching and learning in higher education, presenting evidence for how LXD can be deployed iteratively, rapidly, and thoughtfully. By first identifying what students need, we can then attempt to create LXD solutions that provide affordances to support the needs of student learners in a strategic manner. We assert an alternative approach to synchronous Zoom teleconferenced lectures by detailing the efforts toward designing an asynchronous self-paced online course, offering a pathway for students to further develop their motivations and their learning behaviors in online environments. With 67.4% of students in this study reporting that the knowledge they gain by taking this course can be applied in many different situations (See Fig. two), these learning behaviors may be transferable, and can certainly be utilized in their other courses as students continue distance learning. Fostering students’ learning behaviors such as usage of elaboration and critical thinking are considered key competencies and transferable skills for STEM undergraduate students at R1 institutions linking course materials to real-world practice (Chiaburu & Marinova, 2005; Fries et al., 2020; Halpern, 1998). Moreover, this study may further support the growing literature on learning experience design in higher education courses by drawing on multiple learning design principles, adopting digital learning tools, and user experience design facets intended to enhance students’ online learning experiences through empathy (Kafai, 2005), informing designers as well as instructors on how we might effectively improve asynchronous self-paced online teaching and learning of STEM subjects at the R1 institutional level.
